# Age-related changes in hand dominance and functional asymmetry in older adults

**DOI:** 10.1371/journal.pone.0177845

**Published:** 2017-05-30

**Authors:** Anna Sebastjan, Anna Skrzek, Zofia Ignasiak, Teresa Sławińska

**Affiliations:** 1 Faculty of Physical Education, University School of Physical Education in Wroclaw, Wroclaw, Poland; 2 Faculty of Physiotherapy, University School of Physical Education in Wroclaw, Wroclaw, Poland; University of Ottawa, CANADA

## Abstract

The aim of the study was to investigate fine motor performance and ascertain age-related changes in laterality between the dominant and non-dominant hand. A representative sample of 635 adults (144 males and 491 females) aged 50 years and over completed a test battery MLS (Motor Performance Series) to assess a broad range of hand functions. Functional asymmetry was observed in all four motor tests (postural tremor, aiming, tapping, and inserting long pins). Significant differences between the dominant and non-dominant hand were obtained in both sexes across all age groups, except in the oldest female group (age >70) for the aiming (number of hits and errors) and postural tremor (number of errors) tasks. These differences in age-related changes may be attributed to hemispheric asymmetry, environmental factors, or use-dependent plasticity. Conflicting evidence in the literature warrants additional research to better explain age-related alterations of hand dominance and manual performance in old age.

## Introduction

The upper extremities, specifically the hands, play an active and vital role in daily life. The hands are subject to numerous age-related physiological and anatomical changes that can lead to diminished function [1]. These include local and global degenerative effects related to pathological conditions, deficiencies in skeletal muscle mass and structure, and reduced neural control affecting fine motor performance [2, 3, 4]. Natural involution processes are linked to the progressive decrease in cognitive and sensorimotor functions. This has an undeniable impact on quality of life in elderly people [5, 6]. During the last few years, with the aid of methods such as fMRI, a number of neuroanatomical changes in the nervous system have been observed such as the loss of brain tissue [7], reduction of white and gray matter [8,9], problems with synthesis of dopamine, serotonin, and acetylcholine [10], and changes in the size of the corpus callosum [11]. Another important issue concerns the processes of cerebral metabolism. The weakening of these processes is closely related to changes in mitochondrial dysfunction [12,13], and the prefrontal lobe [14,15]. Changes within different structures or physiology of the brain translate into disorders that can affect upper limb function. Completing a given task by hand in the elderly population requires the activation of more regions in the brain. It is also associated with increased reaction time and decreased coordination and manipulation. Finally, it differs considerably depending on a given task [16, 17, 18]. Changes in the nervous system are related to changes in various functions of the hand. Movements become slower, the time to react to and correct the given activity is longer, and problems occur with holding and manipulating the object and completing tasks at different levels of complexity. This, in turn, forces the brain to create compensatory mechanisms in order to prevent further changes. Moreover, the destabilization of hand functions may be an indication of progressing illnesses of the nervous system [1, 6, 16].

Furthermore, there are indications that hand dysfunction may be induced by external factors [19]. While the literature on age-related changes in hand function is abundant, less is known about changes in the level of functional asymmetry with advancing age. Reports do indicate that senescence is associated with overall decreased hand coordination [20] and that changes in sensorimotor performance may significantly reduce quality of life in old age [21, 22, 23]. Nonetheless, the effects of normal aging on inter-hand differences are not fully explained and are worthy of multidisciplinary investigation. It should also be noted that in the current population of older people, the majority declare their right hand as dominant. Without thorough research, we cannot say whether similar processes occur in people who are left handed, and if these changes are the same or greater than in the case of right-handed people [24].

Research on the development of hand laterality (handedness) in children and adults is well established [25, 26, 27] and is defined as the differentiation in performance and preference between one hand (dominant) and the other (non-dominant) [28, 29]. However, there is contradictory evidence in regard to changes in motor asymmetry in old age. The literature reports age-related increases in dominant hand usage [30], attenuation of hand dominance with a shift to ambidexterity [31], or no change at all [32, 33]. Previous studies, specifically by Weller and Latimer-Sayer [30], suggested a shift towards right hand dominance with age. This finding was grounded in superseded theories of aging [34, 35] that have been largely refuted in recent times [36]. Of particular interest are the results published by Kalisch et al. [31], citing the concept of use-dependent plasticity, which suggests that older age and sedentary lifestyles limit the practice-based superiority of the dominant hand and induce lateral equalization.

Several methods have been devised to assess hand laterality, from the subjective (self-rating questionnaires) to the objective (practical tests using standardized equipment) [37, 38, 39, 40, 41]. Many studies are contradictory in terms of size differences (or lack thereof) reported on functional asymmetry in the elderly.

Hence, there is a need for additional investigation on age-related changes in dominant and non-dominant hand function involving healthy patient populations. This will not only enrich treatment approaches and improve quality of life by restoring impaired hand function, but also improve understanding of laterality in old age.

## Aim

The purpose of the study was to investigate the fine motor performance of older age adults (age >50) and ascertain the magnitude of functional asymmetry and its association with age and sex.

## Material and methods

This study was performed under the auspices of the Ministry of Science and Higher Education (Project No. N404 075337) at the Biokinetics Laboratory, supervised by long-standing faculty members from the Department of Biostructure at the University of Physical Education in Wrocław, Poland. The study protocol was approved by the Ethics Committee for Scientific Research at the University of Physical Education in Wrocław, Poland (Feb 18, 2009).

A sample consisting of 635 individuals (144 males and 491 females) from different socioeconomic backgrounds was recruited from the Lower Silesian region of Poland. Participants were included if they were greater than 50 years old, unambiguously self-rated their right hand dominance, were independent in everyday activities, and lacked an upper limb pathology. All participants gave written, informed consent. The sample was dichotomized according to sex and was divided into three age groups (Table 1).

**Table 1:** Age groups.

Age group	Males		Females		Total	
	*N*	%	*n*	%	*n*	%
50–59	43	30	148	30	191	30
60–69	64	44	245	50	309	49
70+	37	26	98	20	135	21
Total	144	23	491	77	635	100

Hand function was quantified using the Motor Performance Series (MLS) test battery as a component of the Vienna Test System (Dr. G. Schuhfried GmbH, Austria). The MLS battery assesses fine motor performance on a test board with various contact surfaces on which subjects perform a series of static and dynamic tasks using a contact pencil. Testing was performed in the afternoon using both the right and left hand. The S3 short form, as adapted by Vasella, was administered. This test is a part of the Vienna Test System, the first version of which was created in 1978 at Schuhfried. VTS tests are used mainly in neuropsychiatry, neurology, pharmacopsychology, rehabilitation, and developmental, sports, and work psychology.

The form (S3—short) we used was carried out without any modification according to the requirements of the user manual. We chose this form due to the short duration of each task. It was also aimed at fully efficient performance which would preclude any signs of fatigue that might affect the results.

**Fig 1:**
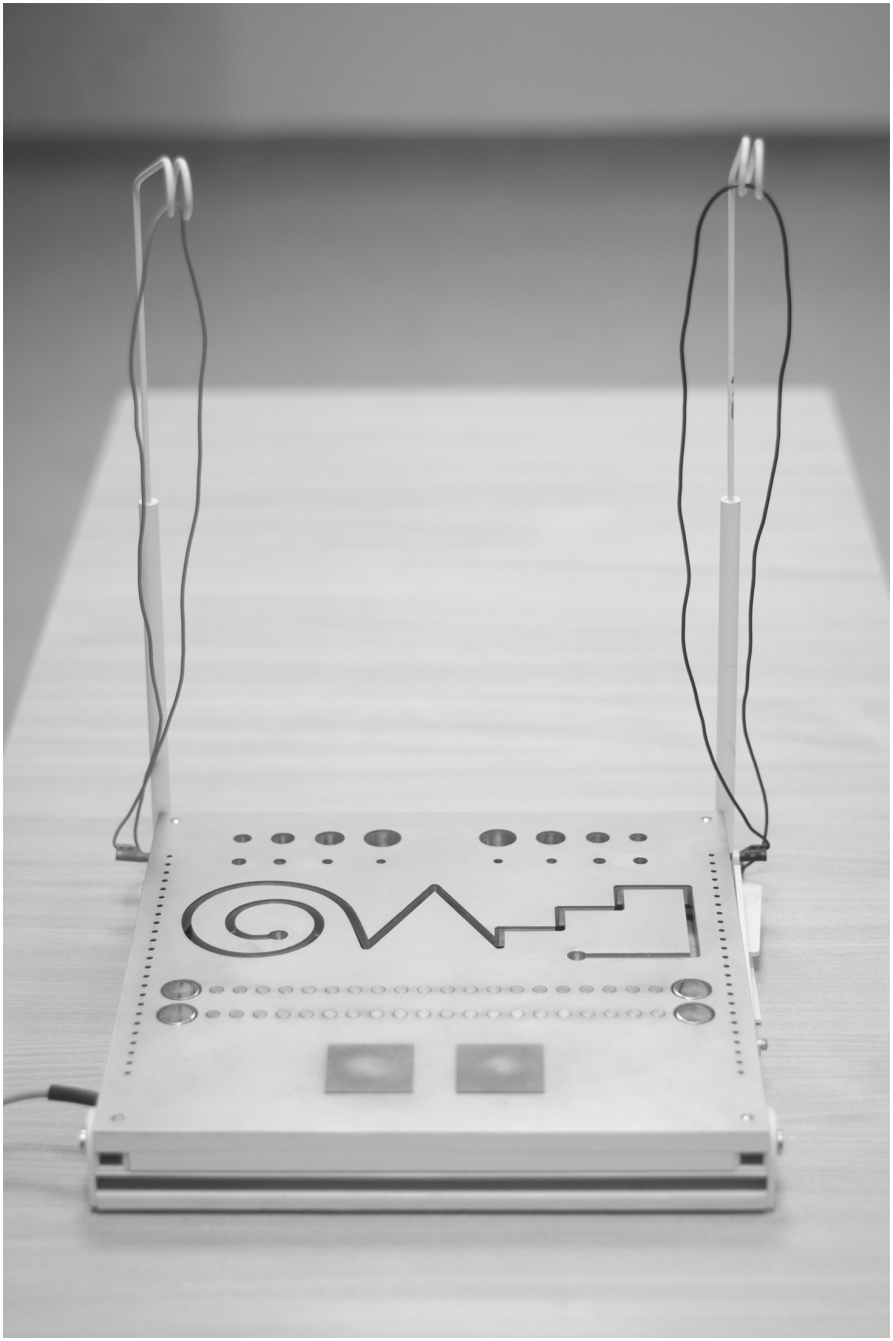
The MLS work board.

The main work board for the MLS battery is a working panel (dimensions 300x300x15mm) with specific surfaces, mills and contact surfaces. These surfaces make it possible to carry out very specific subtests (Fig 1).

On both the left and right side of the work board are test contact pens connected to the board. The test pens are integral with each test. On the right side, there is a black contact pen for the right hand, and on the left side, there is a red contact pen for the left hand.

Prior to beginning the test, each participant declares their dominant hand.

Every time the MLS battery is performed, special attention should be paid to the position of the hand being tested. Participants should not put their hand on the table top or on the work board. Contact pens should be held like any other writing instrument.

Before performing each task, the participant is given instructions on how to complete the task. To perform each test, the participant activates three muscle groups: postural stabilizers, muscles supporting the execution of movement, and the muscles responsible for the execution of movement (forearm and hand). Reliability and description of the test have been confirmed using the test-retest method by Raczek et al. [42].

**Fig 2:**
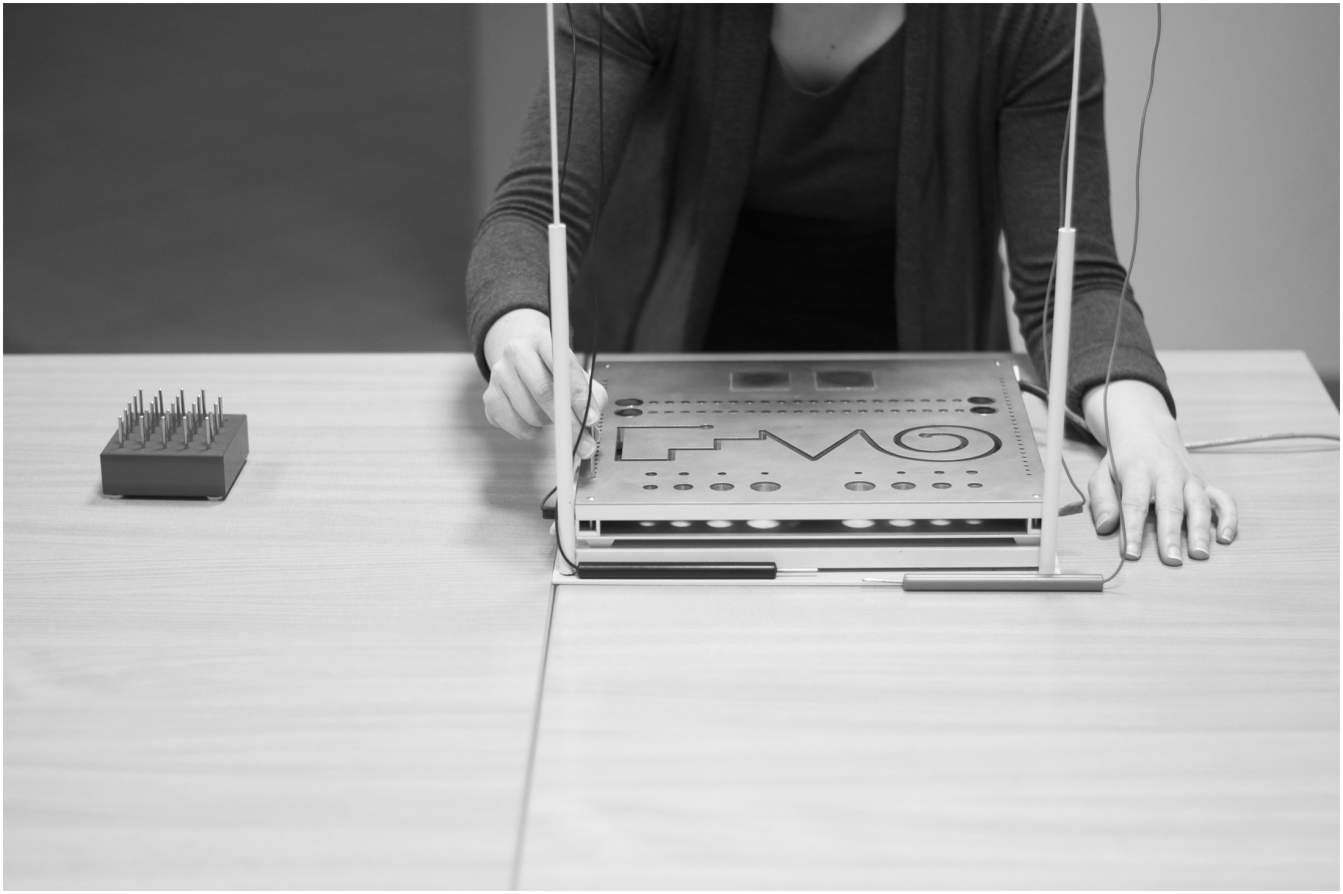
“Inserting Long Pins” task on the MLS test board.

Four out of the five tasks on the battery utilizing both hands were selected for our research:

1. “Steadiness”–the participant places the contact pen vertically in a hole with a diameter of 5.8 mm (the third largest hole in the lower row, on the right side for the right hand and the same hole on the left side for the left hand). It is important for the contact pen not to touch the side wall or the bottom of the hole. The run time of the test is 32 seconds. “Steadiness” measures the ability to maintain a precise and steady position of the arm and hand over a prescribed period of time and is evaluated by the number of errors (contacts made with the test board indicating hand tremor).
2. “Aiming”—using the contact pen, the participant touches 20 points (5 mm in diameter and 4 mm apart) from right to left (for the right hand), or from left to right (for the left hand) as quickly and as accurately as possible. “Aiming” measures hand precision by evaluating the time to consecutively hit a row of target and the number of correct hits and errors (missed targets).
3. “Tapping”—using the end of the contact pen, the participant taps a 40 x 40 mm square plaque as fast as possible for 32 seconds. On the right side, there is a square plaque for the right hand and the same on the left side for the left hand. “Tapping” measures the ability to perform repetitive movements with the wrist and fingers by tapping with the contact pencil as fast as possible. “Tapping” is evaluated by the number of hits in 32 seconds.
4. “Inserting Long Pins”—the participant pulls out 25 pins, one by one, as fast as possible from a container placed at a distance of 30 cm from the work board. The order of pulling is not important. The pins are subsequently placed into 25 holes along the edge of the work board from top to bottom (for the right hand on the right side, for the left hand on the left side; then, the container with pins is placed 30 cm away from the left side of the work board). “Inserting Long Pins” assesses hand and finger dexterity and is measured by the time needed to insert a row of pins (Fig 2).

## Statistical analyses

The distribution of the data set was screened for normality using the Shapiro—Wilk test. Descriptive statistics were calculated for all six task variables. These included the mean, standard deviation [SD], and coefficient of variation for parametric data, the median and interquartile range for non-parametric data, and the minimum and maximum values for each. Multivariate analysis of variance (ANOVA) examined the effects of sex and age with a repeated measures design for the dominant (right) and non-dominant (left) hand as a measure of functional asymmetry. Post-hoc comparisons were performed with Fisher's Least Significant Difference (LSD) method when significant interactions were observed. Non-parametric data was analyzed using the Kruskal—Wallis test for multiple comparisons, and the median test. The Wilcoxon signed-rank test was used to compare the differences between the dominant and non-dominant hand. Statistical significance was accepted at *p* < 0.05 for all statistical procedures. Data processing was performed using the Statistica 10.0 software package (Statsoft, USA).

## Results

The basic characteristics of these functions for males and females in three successive age groups are reflected in Table 2 (the parameters of the normal distribution) and Table 3 (the parameters of the distribution deviating from normal).

**Table 2:** Statistical characteristics parameters of precise motor hand skills in gender and age groups.

Test measure	Sex	Age group	Dominant hand				Non-dominant hand			
			$\bar{x}$	SD	min	max	$\bar{x}$	SD	min	max
Aiming—number of hits [number]	Males	50–59	19,81	0,88	16,00	22,00	20,42	1,01	19,00	23,00
		60–69	19,98	0,77	17,00	24,00	20,39	0,97	18,00	24,00
		70 +	20,22	0,75	19,00	23,00	20,81	1,27	19,00	25,00
	Females	50–59	19,99	0,81	16,00	24,00	20,25	0,99	17,00	24,00
		60–69	20,05	0,75	16,00	23,00	20,43	1,06	16,00	25,00
		70 +	20,20	1,01	17,00	25,00	20,39	1,09	17,00	24,00
Aiming—Total time [seconds]	Males	50–59	8,81	1,67	6,28	12,90	9,27	1,74	2,12	13,04
		60–69	9,58	2,57	6,28	21,00	10,08	2,10	6,47	17,88
		70 +	11,60	3,44	6,90	24,93	11,57	2,87	6,61	19,09
	Females	50–59	9,41	2,08	5,79	16,54	9,93	2,40	0,48	20,44
		60–69	9,99	2,23	3,17	24,95	10,77	2,55	1,03	25,92
		70 +	11,98	3,10	3,00	22,31	12,66	2,70	7,89	24,00
Inserting Long Pins—total time [seconds]	Males	50–59	43,34	4,19	35,74	54,05	46,53	5,43	33,14	58,14
		60–69	45,90	6,60	35,35	66,07	49,50	7,05	37,98	69,19
		70 +	50,30	6,57	35,45	67,48	55,05	7,39	41,15	69,35
	Females	50–59	42,07	5,13	33,56	64,51	47,64	6,90	37,03	74,73
		60–69	43,76	5,18	29,80	60,57	48,99	5,58	36,62	65,43
		70 +	50,52	8,22	38,41	73,07	56,73	9,72	36,36	84,46
Tapping—number of hits [number]	Males	50–59	199,81	24,08	112,00	253,00	186,93	21,80	120,00	229,00
		60–69	193,50	23,33	112,00	241,00	181,59	24,00	131,00	224,00
		70 +	176,92	27,03	100,00	227,00	167,51	25,15	96,00	204,00
	Females	50–59	188,18	22,68	100,00	236,00	171,36	19,91	124,00	229,00
		60–69	181,22	20,27	100,00	230,00	164,47	17,79	116,00	225,00
		70 +	168,46	20,51	121,00	223,00	155,68	19,67	101,00	210,00

**Table 3:** Statistical characteristics parameters of precise motor hand skills in gender and age groups—non-parametric tests.

Test measure	Sex	Age group	Dominant hand					Non-dominant hand				
			median	lower quartile	upper quartile	min	max	median	lower quartile	upper quartile	min	max
Aiming—number of errors [number]	M	50–59	0,00	0,00	1,00	0,00	8,00	1,00	0,00	3,00	0,00	11,00
		60–69	0,00	0,00	2,00	0,00	7,00	1,50	0,00	3,50	0,00	13,00
		70 +	0,00	0,00	2,00	0,00	7,00	1,00	0,00	4,00	0,00	9,00
	F	50–59	0,00	0,00	1,00	0,00	15,00	1,00	0,00	2,00	0,00	12,00
		60–69	0,00	0,00	1,00	0,00	14,00	1,00	0,00	2,00	0,00	12,00
		70 +	0,00	0,00	1,00	0,00	15,00	1,00	0,00	2,00	0,00	13,00
Steadiness—number of errors [number]	M	50–59	0,00	0,00	2,00	0,00	10,00	1,00	0,00	4,00	0,00	19,00
		60–69	0,00	0,00	1,50	0,00	20,00	1,00	0,00	2,00	0,00	23,00
		70 +	0,00	0,00	3,00	0,00	25,00	1,00	0,00	10,00	0,00	39,00
	F	50–59	0,00	0,00	1,00	0,00	20,00	0,00	0,00	2,00	0,00	21,00
		60–69	0,00	0,00	2,00	0,00	29,00	1,00	0,00	3,00	0,00	39,00
		70 +	1,00	0,00	5,00	0,00	32,00	2,00	0,00	6,00	0,00	32,00

M—males, F—females

Significant differences in hand function were registered in the four tasks for sex, age, and hand asymmetry (Tables 4 and 5).

**Table 4:** Main effects of age, sex, and hand asymmetry (dominant vs. non-dominant) on test variables.

Test variable	Effect							
	Hand asymmetry		Hand asymmetry × sex		Hand asymmetry × age		Hand asymmetry × sex × age	
	*F*	*p*	*F*	*P*	*F*	*p*	*F*	*P*
Aiming—number of hits [number]	44.78	**0.0000**	4.59	**0.0325**	0.06	0.9458	1.11	0.3315
Aiming—total time [seconds]	14.16	**0.0002**	1.88	0.1714	0.53	0.5888	0.47	0.6250
Tapping—number of hits [number]	188.48	**0.0000**	4.29	**0.0388**	1.25	0.2878	0.05	0.9479
Inserting Long Pins—total time [seconds]	314.76	**0.0000**	11.56	**0.0007**	1.59	0.2049	0.26	0.7706

Significant differences (*p* ≤ 0.05) denoted in bold

**Table 5:** Parametric comparisons of dominant and non-dominant hand test scores (excluding number of errors) for sex and age.

Test variable	*p* values for between-hand differences					
	Males			Females		
	50–59 years	60–69 years	70+ years	50–59 years	60–69 years	70+ years
Aiming—number of hits [number]	**0.0014**	**0.0086**	**0.0035**	**0.0095**	**0.0000**	0.1407
Aiming—total time [seconds]	0.2471	0.1319	0.9399	**0.0165**	**0.0000**	**0.0107**
Tapping—number of hits [number]	**0.0000**	**0.0000**	**0.0042**	**0.0000**	**0.0000**	**0.0000**
Inserting long pins—total time [seconds]	**0.0001**	**0.0000**	**0.0000**	**0.0000**	**0.0000**	**0.0000**

Significant differences (*p* ≤ 0.05) denoted in bold

In the “Aiming” task, strong asymmetry between the dominant and non-dominant hand was observed in both the number of hits and time to completion. More detailed comparisons (Table 5) revealed that the number of hits was significantly different between both hands in all three male age groups and two female age groups (excluding those aged >70 years). According to the description of the test, any result above or below 20 hits is treated as incorrect. With increased age, both for males and females, better results were observed in the non-dominant hand as compared to the dominant hand. The time to completion was significantly different between both hands only in females (all age groups). Females required more time to perform this test with the non-dominant hand, suggesting that their movements were significantly slowed.

Strong functional asymmetry was also evident in the “Tapping” (number of hits) and “Inserting Long Pins” (time to completion) tasks. Here, significant differences between the hands were observed in both the females and males across all age groups (Table 5). The higher the participants’ age, the more time they needed to complete the task "Inserting Long Pins". These findings suggest that hand and finger dexterity decrease with age. In the first two age groups, the average results did not vary widely for both hands in either sex, although we observed a significant increase in the time needed to complete the test. In the two oldest age groups the total time increased significantly. Greater age was associated with slower test times in both females and males. Males completed this task more quickly than females, both in the dominant and non-dominant hand. In the "Tapping" test males also performed better, completing a larger number of taps within 32 seconds than females in every age group and in both upper limbs. In this test, we observed a decreased ability to perform repetitive movements with the wrist and fingers in all of the female groups.

Testing for normality revealed abnormally distributed data for the number of errors in the “Aiming” and “Steadiness” tasks. In Table 6, only the results from participants who committed errors in these two tests are shown. Therefore, the number of participants in Table 6 differs from the number of participants in other tables.

**Table 6:** Non-parametric comparisons of dominant and non-dominant hand test scores (number of errors in “Aiming” and “Steadiness” tasks) for sex and age.

Test variable	Age group	*n*	Females			*N*	Males		
			*T*	*Z*	*p*		*T*	*Z*	*p*
Aiming—number of errors [number]	50–59	104	1231.00	4.86	**0.0000**	26	96.00	2.02	**0.0435**
	60–69	162	3101.00	5.85	**0.0000**	44	175.00	3.73	**0.0002**
	70+	58	673.50	1.41	0.1588	29	82.00	2.93	**0.0034**
Steadiness—number of errors [number]	50–59	81	1029.50	2.97	**0.0030**	23	98.50	1.20	0.2296
	60–69	151	3956.00	3.31	**0.0009**	43	285.50	2.26	**0.0236**
	70+	72	1017.00	1.67	0.0956	23	64.50	2.24	**0.0254**

Significant differences (*p* ≤ 0.05) denoted in bold

Non-parametric testing indicated significant differences between the dominant and non-dominant hand in the “Aiming” task for all male and female age groups except females aged >70 years (Table 6). In “Steadiness”, significant differences indicative of functional asymmetry were observed in males aged 60–69 and >70 years and in females aged 50–59 years and 60–69 years (Table 6). On average, females made less errors than males in the "Aiming" test and had much better hand precision. However, males had a better ability to maintain precise and steady positions of the arm and hand over a prescribed period of time.

## Discussion

While age-related changes in hand function have been well described in the literature [43, 44, 45, 46], this study analyzed the ambiguities still surrounding changes in hand dominance (laterality) with increasing age. A review of the available findings suggests a number of explanations for shifts in hand dominance and variation of functional asymmetry with aging.

According to some hypotheses, with age, we can observe significant and important advantages of the dominant hand over the non-dominant hand. Citing the incidence of right hand dominance in old age, studies argue that the right hemisphere shows greater age-related decline than the left hemisphere, thereby inducing asymmetry [34, 35, 47, 48]. This was historically the most popular model in terms of explaining this basic phenomenon. However, recent studies have suggested that there is a reduction in the asymmetric aging of the hemispheres, which explains the lack of significant and relevant differences between the dominant and non-dominant hands found in other studies [32, 49]. Another alternative posits the concept of use-dependent plasticity [31], which coincides in part with the previous explanation.

There are a few limitations in our research. First, there was no random selection so we are unable to treat our results as representative of the elderly population as a whole, despite our large sample size of 635 participants. Second, due the difficulty of finding participants who fit our inclusion criteria, specifically participants >70 years of age, some of the groups were less numerous. This may have impacted our results as our research shows that women >70 years of age are particularly exposed to these major changes. We did not notice such tendencies in men. This may be related to the size of the groups, particularly the oldest groups. Third, we did not examine other factors that may influence functional asymmetry, due to the large number of such factors that exist. We also know that not using well-known questionnaires with all our participants may have limited our findings, but we wanted to get both a subjective and objective analysis from our patients to give a better representation of their functional asymmetry. Finally, as our cross-sectional research demonstrates only some trends in hand dominance, we cannot say how it may look in longitudinal data. But even the tendencies found in our study can be very useful when looking at the elderly and the environment.

In our research, we observed variations in the magnitude of functional asymmetry that depended on the test variable that was studied. The largest differences between the dominant and non-dominant hands (in both males and females across all age groups) were in the “Inserting Long Pins” (hand and finger speed and agility) and “Tapping” (wrist and finger speed) tasks. Statistically significant asymmetry was not observed in the majority of the remaining test variables. This may indicate a reduction in hemispheric asymmetry or attenuation of dominant hand superiority, possibly due to its overexploitation in earlier life. Substantiated conclusions are difficult to draw considering the conflicting neurophysiological data on the subject [50, 51]. In clinical fMRI, in people divided into two age groups (younger and older), while performing tasks and at rest there were observable differences in the response of different regions of the brain to the task [32]. In the elderly, there is not only activation of the motor cortex while performing motor tasks, but also activation of regions engaging in information processing supply and participating in the planning of movement, time control, and coordination. In an aging brain engaging in such tasks, both hemispheres are involved. Results of other studies indicate that in the elderly, compensation of disorders in the nervous system are a result of decrease in brain volume and changes in brain metabolism and blood flow in the brain [52]. Elderly people engage more brain regions than their younger counterparts when performing a single task. Recent studies also indicate that these changes in dexterity are related to modifications of the corpus callosum in the brain that occur across the entire life span [11]. The corpus callosum is responsible for the transmission of information between the right and left hemispheres. Changes in its size [53] or structure [54, 55] may have an effect on the differences in interhemispheric communication and inhibition, and affect the reorientation of functional asymmetry especially in comparisons of young adults and older adults [11, 53, 54, 55]. Involution changes in the aging brain may lead to a reorganization of the use of the upper limbs. We suggest that perhaps the reduction of functional asymmetry is a completely natural process of the brain and other structures adapting to changing opportunities to use the upper limbs to perform a given task.

At the same time, the results of our research allow us to conclude that more pronounced changes are observed in precise motor skills in females in comparison to males, especially in tests where the evaluated parameter was the total time for task completion. Females were more accurate in their performance of single tasks which can be correlated with the increased time needed to complete each task. We postulate that perhaps the significant decrease in the performance of specific tasks by females is associated with their roles in family and social life, which in their younger years involves performing many everyday activities using their upper limbs, translating into a greater decrease in their function as they age.

Similar to the present study, Kalisch et al. [31] administered the MLS test battery and a self-rated questionnaire to a sample of 20- to 90-year olds. This study found a greater decline in dominant hand function with age, with performance approaching the level of the non-dominant hand. Surprisingly, the older participants declared themselves to be unambiguous right-handers regardless of the attenuation of dominant hand performance. Kalisch et al. [31] credited these changes to how the dominant hand loses its practice-based advantage in activities of daily living over time. With retirement, increased sedentary behavior, and age-related limitations, older adults no longer maintain the intensity and load that once favored the dominant arm. The results obtained by our study, as well as by Kalisch et al. [31], confirm this decrease in functional asymmetry. What is more pertinent is that the same research tool was used in both studies. The difference between the two studies is the comparison of adults and the elderly in our study, while Kalisch et al. also included a younger group of participants (20 years of age).

The same results were obtained in research done by Przybyła et al. [56] and Sivagnanasunderam et al. [57]. Furthermore, Sivagnanasunderam et al. [57] showed that age-related changes in lateral asymmetry are influenced in large part by the type of administered tasks used to measure hand function. This is confirmed by our results, where differences in functional asymmetry were also observed and were dependent on the type of task performed. This shows that the difference in performance is less marked in complex tasks in comparison to simple tasks. At the same time, both in our study and in the above-mentioned studies, we indicate as a limitation that there are no cross-sectional studies which could confirm our findings or other research findings.

In contrast, Bowden and McNulty [58] recruited 70 individuals (35 females and 35 males aged 20–88 years) free of neurological symptoms. A tapping task and grooved-pegboard task (similar to the “Inserting Long Pins” subtest) revealed no age-related changes in the magnitude of functional asymmetry. Changes in hand lateralization oscillated at 3–10% among the age groups in both the tapping and grooved-pegboard tasks and were not significant. However, males and females performed better with the dominant hand than the non-dominant hand in all age groups.

Gonzales et al. [59] investigated how handedness changes across the lifespan. The subjects were divided into 3 age groups: 2 to 11 years old (80 participants), 17 to 28 years old (25 participants), and 57 to 90 years old (16 participants). They used the modified versions of the Edinburgh and Waterloo handedness questionnaires and two tasks: grasp-to-eat and grasp-to construct. There were no differences between the age groups in relation to hand preference. They also showed more frequent use of the right hand in the grasp-to-eat task than the grasp-to-construct task, which required the use of both hands. Conclusions made by Gonzales et al. acknowledged that their small number of participants does not allow them to say with certainty that this trend of changes represents society as a whole. In addition, studies have not been longitudinal so we can only hypothetically draw conclusions regarding the trends of changes in functional asymmetry with age. Gonzales and Bruin [60] investigated the spatial abilities that allow us to visually locate an object in space to understand the two- and three-dimensional (2D and 3D) relationships between objects and our environment. Spatial abilities help us to safely navigate our environment and are very important for a person to be functionally independent. Spatial ability is a very complex process, with many components that require individual action to allow for successful completion of a spatial task [61, 62]. One of these components is the mental rotation ability that allows us to perform everyday activities like combing hair or preparing meals. The study by Bruin and Gonzales [60] utilized 44 right-handed participants (females and males) who were divided into two age groups: younger adults (18–25 years old) and older adults (60–81 years old). In this study, older adults demonstrated a substantial and statistically significant advantage of right, dominant hand use. This applied to both the capture the individual building block task and to building 2D and 3D models. The results of both studies confirmed the hypothesis of left hemisphere specialization for visually-guided actions, and that mental rotation is primarily a right-hemisphere specialization. Interestingly, it appeared to be more specific to older adults. However, the authors emphasized the need for further research related to this process in order to investigate additional explanations for the observed changes.

Additionally, findings by Paizis et al. [63], suggest that there are no changes in functional asymmetry with age. Interestingly, lateralization completely disappeared in older adults while mental actions showed no statistically significant difference for the time of execution of mental actions in both groups. The results obtained by this study differ from ours, which may be associated with the number of participants in each group, the division of age and gender per age group, or growing age differences between the right dominant and left non-dominant hand.

However, almost every researcher whose research has been in contradiction of other studies has suggested that there needs to be further in-depth analysis of this phenomenon. Perhaps the impact on the results observed in our study, along with other studies, is related to age groups, the number of participants tested in a given group, the specifics of the tools and test used, the environment, education level, or the absence or presence of coexisting illnesses. We did utilize a large questionnaire to ensure our participants did not have an illness that may be connected with right hand use. Not all researchers performed this extra step in their research.

Nonetheless, the findings of the present study and those by Kalisch et al. [31] do suggest that age-related changes in manual laterality and performance may be related to the use of both hands with equal frequency in older age. The strong associations between fine motor skills and physical fitness as reported in the literature also support this reasoning [1, 64, 65, 66]. Other studies have examined the role of environmental and social factors. Among others, Skrzek et al. [19] investigated fine motor performance of the dominant and non-dominant hand with regard to lifestyle in 486 females dichotomized as active and sedentary. They observed a decrease in functional asymmetry with age regardless of lifestyle, with the decline in performance greater in the dominant hand, giving evidence for age-related lateral equalization. This finding suggests that an active lifestyle by itself may not necessarily affect lateral asymmetry in older age, contrasting to a degree the conclusions drawn by Kalisch et al. [31]. However, Skrzek et al. did state that current neurophysiological knowledge is not yet definitive in regards to the concept of use-dependent plasticity.

In summary, our results show that gender, age, and lateralization have different effects on specific coordination skills in the field of precise hand motor skills. In most tests, females had better results that were statistically significant in different age groups, especially in the dominant upper limb.

In both studied groups (males and females), the greatest differences were found in the comparisons between the youngest and the oldest age groups, and between the 60–70 year old age groups. Evolutionary changes are much more pronounced in females than in males when comparing age groups, showing statistically significant differences.

Lateralization is very clearly observed in the studied parameters. The majority of them show statistically significant differences in laterality in all age groups in both males and females. This diversity sometimes loses its relevance. It is visible in females in the oldest groups (>70 years of age) in three studies: the test of aiming (number of hits, number of errors) and tremor (number of errors).

Differences in the reports of other authors on functional asymmetry (decrease, increase, constant level differences) may be associated with correlations between different factors, the strength of their mutual relations, or external factors such as community roles—the duties that older people performed in their communities at a younger age.

Furthermore, in our research we have confirmed the undisputed claim that age determines changes in hand function. It seems to us that our results are interesting due to the fact that both adults and older people were considered and that we ensured our participants were healthy in terms of diseases that may affect the performance of manual tasks. Differences in inter-group comparisons between younger and older participants are obvious. In our research, we show at what moment the changes become visible and substantial. At the same time, we show that the body at this stage is able to compensate for some of these changes by reducing functional asymmetry. In addition, we believe that the social roles that young people play in their environment can influence these results as they age.

As expounded in the present study, changes in hand dominance and the modulation of functional asymmetry with advancing age are still ultimately unexplained and therefore warrant further investigation. Research should address the fact that studies examining laterality and hand dominance overwhelmingly involve self-declared right-handers (70–95%) which, due to cultural and environmental pressures against left-handers, may have distorted results [24]. For this and other reasons, it is difficult to identify the mechanisms responsible for the observed changes without relevant data from individuals confirmed to be unambiguous left-handers.

## Conclusions

1. Significant hand laterality was observed in the tasks quantifying fine motor performance across all age groups and both sexes.
2. Visible trends of change, despite our ambiguous results, suggest that we can observe attenuation of dominant hand superiority in task performance as age increases.
3. Further and extended analysis is required to determine if the age-related alterations of hand dominance and manual performance in old age are attributed to hemispheric asymmetry, environmental factors, use-dependent plasticity or use-dependent receptor loss. Hand Therapy must focus on the interaction between social, functional, physiological and biomechanical elements. Before hand therapy is implemented, it must be based on accurate observations and evaluations of individual elements of hand function, including changes in functional asymmetry. Any tasks given to elderly people should take into account a decrease in hand function asymmetry, which raises the issue of uniform stimulation of both hands.
